# Intestinal Incarceration and Strangulation by the Median Ligament of the Urinary Bladder in a Dog

**DOI:** 10.3390/ani14223265

**Published:** 2024-11-13

**Authors:** Luke Ellis, Arthur House

**Affiliations:** Peninsula Vet Care, Emergency and Referral Hospital, Mornington, VIC 3931, Australia; arthur@penvetreferral.com.au

**Keywords:** canine intestinal strangulation, canine intestinal incarceration, jejunal strangulation, median ligament urinary bladder

## Abstract

A 4.5-year-old female spayed, mixed-breed (Beagle X Cavalier King Charles Spaniel) presented with acute onset of vomiting and abdominal pain. The dog had undergone an exploratory celiotomy for comparable symptoms 9 months earlier, with no aetiology identified. Computed tomography (CT) was highly suggestive of mechanical obstruction of the small intestine. An exploratory celiotomy was performed and identified strangulation of the jejunum by a rent within the median ligament of the urinary bladder. It was hypothesised the rent formed secondary to previous surgical trauma. Complete excision of the median ligament resulted in resolution of clinical signs. This report emphasises the importance of minimising the formation of mesenteric rents when performing abdominal surgery.

## Simple Summary

In this study we present the case of a dog that presented with vomiting and abdominal pain. Computed tomography investigation was indicative of a mechanical obstruction, and surgical exploration of the abdominal cavity revealed strangulation of the jejunum by a rent in the median ligament of the urinary bladder. It is hypothesised this rent was created during previous surgical exploration. Excision of the median ligament resolved the strangulation and obstruction of the affected jejunum and resulted in complete resolution of clinical signs.

## 1. Introduction

An acute abdomen typically presents as sudden onset abdominal pain with nausea or vomiting. Multiple aetiologies can cause these nonspecific clinical symptoms, including infection, inflammation, vascular occlusion, and obstruction [1]. Depending on the underlying cause, diagnosis before surgical exploration can be challenging, and clinical symptoms can be rapidly progressive.

Entrapment of the intestines results in abnormal anatomical positioning within the abdomen cavity, which can result in impeded function due to luminal obstruction. Progression to incarceration and strangulation of the displaced intestines can occur. Compared to simple intra-luminal obstructions, vascular compromise of strangulated intestines can progress rapidly, resulting in ischemia, septic shock, or endotoxic shock, and hence, timely stabilisation and surgery are indicated [2]. In dogs, extraluminal incarceration and strangulation is a rare condition, with reports limited to body wall hernias, omental tears, mesenteric rents, intra-abdominal lipomas and adhesions, and following duodenocolic ligament rupture [3,4,5,6,7]. To the authors’ knowledge, this is the first case report that describes intestinal incarceration and strangulation by the median ligament of the urinary bladder in a dog.

## 2. Case Description

A 4.5-year-old female spayed mixed-breed (Beagle X Cavalier King Charles Spaniel) dog was referred to a private veterinary emergency service with a 24 h history of hyporexia, vomiting, dyschezia, restlessness, and abdominal pain. The patient had presented with similar symptoms 9 months earlier and had responded to supportive care after an unremarkable exploratory laparotomy. Prior to referral, supportive care was initiated, and an AFAST abdominal ultrasound was performed, noting mild enlargement of jejunal lymph nodes and a small volume of free fluid in the cytosolic window.

**Figure 1:**
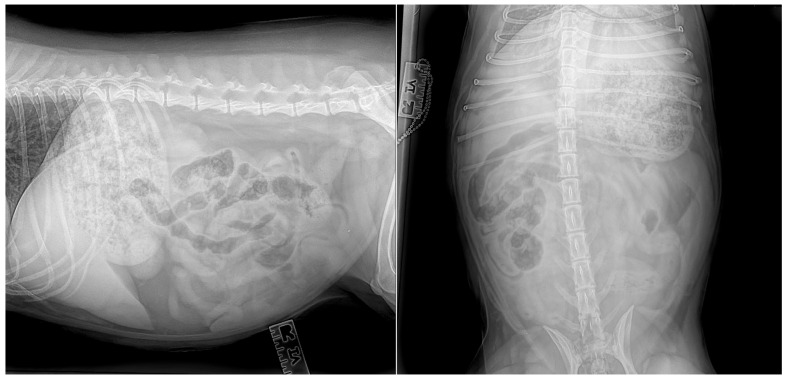
Abdominal radiographs revealed dilated intestines and mixed heterogeneous gas and soft tissue attenuating material within the stomach without evidence of a foreign body or obstructive pattern.

On presentation, the dog was mildly depressed, weighed 20.3 kg, and had a body condition of 6/9. Her rectal temperature was 37.7 degrees, her heart rate was 100 beats per minute, and her respiratory rate was 32 breaths per minute. Her mucous membranes were pink, mildly injected, and moist with normal capillary refill (<1 s). The abdomen was tense, and there was marked pain and vocalisation with mid-abdominal palpation. Packed cell volume and total solids were within normal limits at 43% (37–55%) and 75 g/L (reference range, 55–75 g/L), respectively. A complete blood count (IDEXX ProCyte Dx, Westbrook, ME, USA) and biochemistry (IDEXX Catalyst One, Westbrook, ME, USA) were within normal limits. Venous blood gas analysis (Radiometer, Copenhagen, Denmark) revealed mild hypercapnia (pCO2 44.7, range 34.0–39.0), mild hypokalaemia (3.3, range 3.4–5.3), and mild hyperglycaemia (6.7, range 3.6–6.2). Lactate was within normal limits. Ultrasound-guided abdominocentesis was performed, and the abdominal fluid collected was an acellular high-protein transudate with a total protein of 43 g/L (refractometer). The dog was hospitalised for initial supportive care. An intravenous (IV) catheter was placed, and a lactated ringer solution supplemented with 10 mmol potassium chloride was administered at a rate of 100 mL/h. A fentanyl constant rate infusion was started at 3 ug/kg/h, and maropitant 1 mg/kg was administered IV. Three-view abdominal radiographs were performed to identify a moderate amount of gas and fluid within the stomach and intestines with mild dilation of both organs (Figure 1). No foreign body was identified, and faeces were present within the colon. There were good serosal details, and the findings were not conclusive of an obstructive pattern. Differential diagnoses included radiolucent gastric or intestinal foreign bodies, gastroenteritis, acute pancreatitis, or peritonitis.

As the dog was stable, it was decided to continue supportive care and monitor the clinical response to therapy. Over the next 12 h marked abdominal pain persisted despite intravenous analgesia. The dog was anorexic and had not passed faeces since hospital admission. Hypokalaemia (3.3, range 3.4–5.3), hypercapnia (47.8, range 34–39), and intermittent regurgitation persisted. Free fluid volume on AFAST was static. It was elected to proceed with additional imaging due to a lack of clinical improvement.

**Figure 2:**
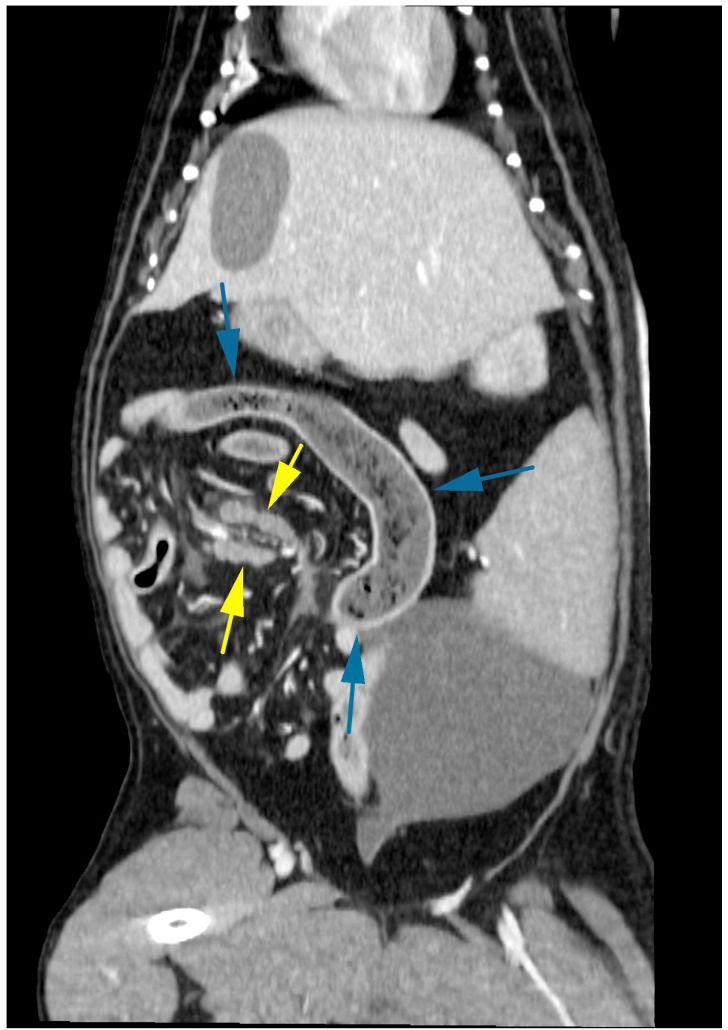
Abdominal CT in the transverse plane revealed a moderately dilated segment of the jejunum with mixed heterogeneous gas and soft tissue opacity (blue arrows), with moderate enlargement of the adjacent lymph nodes (yellow arrows).

**Figure 3:**
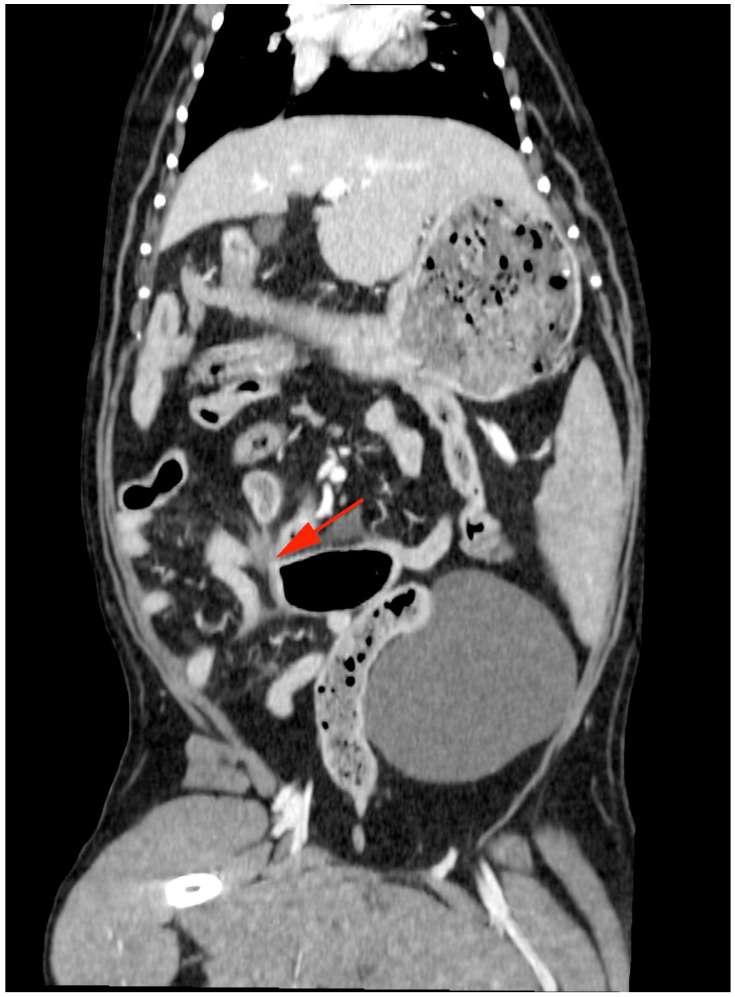
Abdominal CT in the transverse plane displaying the caudal segment of dilated jejunum containing gas and tapering abruptly (red arrow).

The dog was premedicated with a 5 ug/kg IV fentanyl bolus and anaesthesia induced with alfaxalone IV to effect. Anaesthesia was maintained with isoflurane and oxygen. Initial tachycardia was noted after induction, which resolved without medical intervention. Computed tomography (CT) was performed using a 16-slice helical scanner (GE Optima, Chicago, IL, USA) with a slice thickness of 1.25 mm, pitch of 0.938, rotation time of 0.8 s, 120 kVp, and 300 mAs. The patient was positioned in dorsal recumbency, and pre- and post-contrast (2 mL/kg IV iohexol) images of the thorax and abdomen were performed. All CT studies were submitted for review by a board-certified radiologist. There was a long (approximately 14 cm) moderately dilated segment of jejunum, which contained gas and mixed heterogeneous gas and soft tissue attenuating material (Figure 2, blue arrows). The caudal aspect was most significantly dilated, contained more gas, and tapered abruptly (Figure 3, red arrow). There was mild peritoneal effusion and fat stranding. The adjacent lymph nodes were moderately enlarged with an undulating outline and were hypoattenuating (Figure 3, yellow arrows). The stomach was mildly dilated with mixed material, the duodenum and pancreas were unremarkable, and there was a small amount of formed faecal material within the colon. There were no overt signs of an intestinal foreign body.

The imaging findings were suggestive of a mechanical intestinal obstruction. Differential diagnoses included previous surgical adhesions, strictures, or, less likely, intestinal torsion. Considering the imaging findings and the dog’s static clinical condition, it was elected to proceed with an exploratory laparotomy.

**Figure 4:**
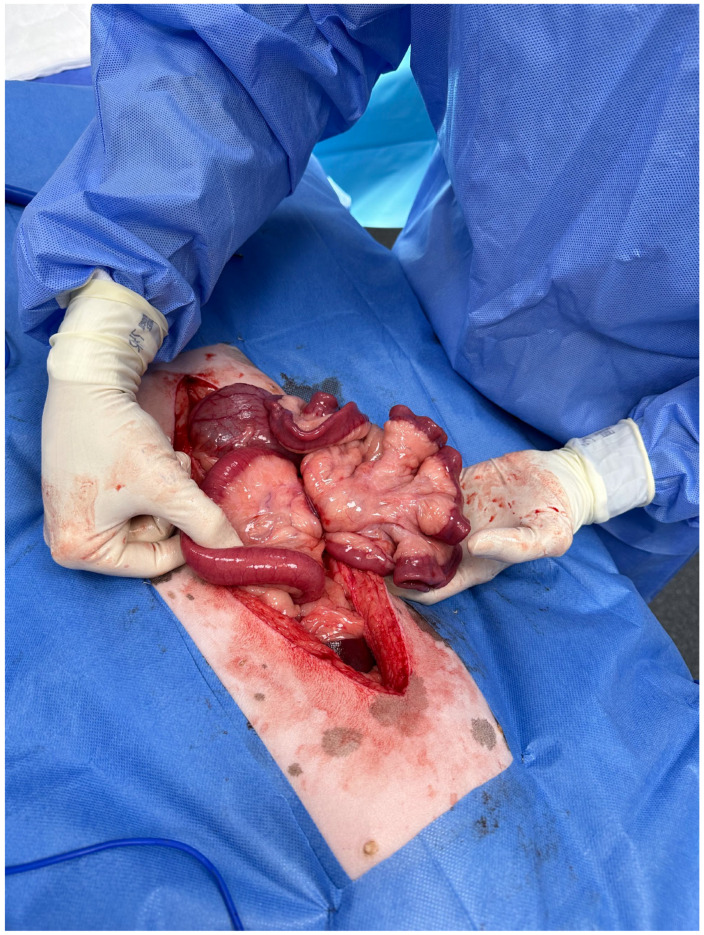
Intraoperative image revealing moderate congestion of the affected jejunal segment and the soft tissue structure strangulating the jejunal mesentery.

**Figure 5:**
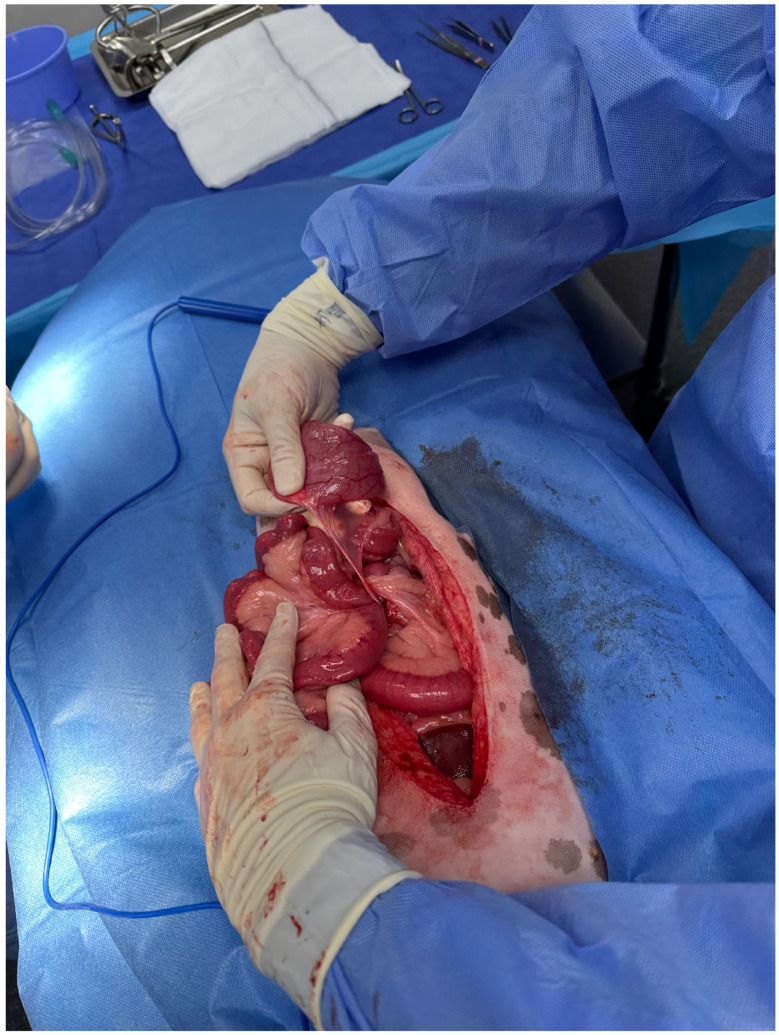
Intraoperative image of the insertion of the ventral median ligament at the apex of the bladder and the jejunum displaced through the rent within the ligament.

Intravenous cefazolin (22 mg/kg) was administered. A ventral midline celiotomy from the xyphoid to the pubis was performed. The falciform fat was excised using monopolar electrocautery. A small amount of serosanguinous free abdominal fluid was present, and a sample was obtained for further analysis pending findings. A routine abdominal examination revealed a homogenous congested dark red region of the jejunum approximately 40 cm long, with partial strangulation of the mesentery by a soft tissue structure (Figure 4). There was no evidence of peristalsis within the affected segment, and palpation of intestinal wall thickness was uniform. There was no evidence of mesenteric torsion. The strangulating tissue was attached caudally to the apex of the bladder and cranially to the linea alba, consistent with the median ligament of the urinary bladder (Figure 5). The ligament was transected at its origin and insertion, and the affected jejunal segment was reduced. Over the next 10 min peristalsis, mild active haemorrhage within the mesentery and improvement of colour (dark red to paler pink) were observed in the affected intestines. The intestinal segment was subjectively deemed viable without evidence of necrosis, and resection was not performed. The rest of the abdomen was examined and deemed unremarkable. The abdomen was flushed with warmed sterile saline, and the lavage was removed with suction. The abdominal wall was closed using 0 PDS in a simple continuous suture pattern. The subcutaneous tissue was closed with 3-0 PDS in a simple continuous pattern. The skin was closed using surgical staples.

Recovery from surgery and anaesthesia was unremarkable. Lactated ringer solution was continued IV at maintenance rates (60 mL/h) post-surgery. A 50 ug transdermal fentanyl patch was placed, and fentanyl constant rate infusion continued and was weaned over 24 h by patient pain assessment. Paracetamol 10 mg/kg IV q8hr was administered postoperatively in the hospital. Venous blood gas analysis (Radiometer) 24 h post-operative was within normal limits. The patient’s demeanour and appetite had markedly improved 24 h post-operatively and was discharged from the hospital approximately 36 h after surgery. Meloxicam was considered post-operatively; however, due to concerns of gastrointestinal side effects, the patient was prescribed paracetamol 250 mg PO q8hr for 3 days post operatively. Follow-up at 2 weeks, 4 months, and 16 months post-operative revealed complete resolution of clinical signs and a good clinical outcome. No further treatment was required for this patient following discharge from the hospital.

## 3. Discussion

Intestinal entrapment, incarceration, and strangulation result in progressive vascular compromise and intestinal obstruction, with both local and systemic consequences [8,9]. Impeded intestinal microvascular perfusion causes local ischaemia and altered intestinal motility. Stasis of fluid within the intestinal lumen acts as an ideal medium for microbial growth and proliferation and, consequently, results in increased bacterial endotoxin production [10]. Translocation of bacteria occurs with a breach of the mucosa, resulting in upregulation of the acute local inflammatory response, which can progress systemically [11]. Untreated strangulation can ultimately result in local tissue necrosis, systemic inflammatory response syndrome (SIRS), multiple organ dysfunction syndrome (MODS), and death [11].

Previously reported cases of intestinal strangulation include intra-abdominal lipomas and adhesions, perineal hernias, an omental tear, and secondary to partial rupture of the duodenocolic ligament [3,4,5,6,7]. This case report describes intestinal incarceration and strangulation by the median ligament of the urinary bladder in a dog. The median ligament connects the urinary bladder to the linea alba and pelvic symphysis. In the foetus, this ligament supports the urachus and persists after birth [12]. The anatomic location of the ligament puts the structure at high risk of damage or rupture when performing a routine celiotomy. The patient had previously presented with an acute abdomen 9 months earlier, with the investigation and an exploratory celiotomy performed. No underlying aetiology was identified, and the patient responded to symptomatic treatment.

Generally, transection of the median ligament is considered benign and associated with minimal clinical consequences [2]. Typically, a complete transection of the median ligament occurs with routine midline abdominal exploration, which increases the exposure of the caudal abdomen and removes the risk of tissue strangulation. Veterinarians commonly aim to reduce morbidity through smaller surgical approaches. When performing a midline celiotomy, a smaller approach also reduces exposure and visualisation of intra-abdominal structures such as the median ligament of the urinary bladder. This potentially increases the risk for partial transection or iatrogenic rent formation within the median ligament compared to celiotomy incisions extending from the xyphoid to the pubis.

The authors hypothesise that the median ligament of the urinary bladder was partially ruptured at the patient’s previous celiotomy but not completely excised. A persistent rent within the ligament was the source of intestinal entrapment and, consequently, incarceration and strangulation.

The typical presentation with strangulated intestine is consistent with an acute abdomen; however, clinical symptoms vary with the degree of vascular compromise and strangulation [2]. While microcirculatory changes within the intestines occur prior to clinical symptoms, the progression of symptoms and irreversible damage to the intestinal wall is often rapid [10]. Resection and anastomosis of non-viable intestines due to strangulation is common [3,4,5,6,7]. In contrast, the dog in this report was stable and initially responsive to supportive treatment with fluids and analgesia. Initial diagnostics included three view abdominal radiographs, which were not conclusive of an obstructive pattern, with a maximal intestinal diameter to midbody fifth lumbar vertebrae ratio of <2.4 [13]. Abdominocentesis of scant-free abdominal fluid was not consistent with a septic abdomen. Hence, the clinicians continued with medical management for 72 h prior to surgical intervention. Computed tomography (CT) has been suggested to be more sensitive than radiographs for identifying mechanical intestinal obstruction [14] and was diagnostic in this case (Figure 3, red arrow). Despite the prolonged duration of strangulation, the incarcerated region of the jejunum was subjectively viable at surgery, with evidence of active haemorrhage and peristalsis [10]. This is likely due to the rent in the ventral median ligament being relatively large and partially impeding vascular perfusion of the affected jejunal segment.

## 4. Conclusions

This case report describes the successful management of intestinal strangulation by the median ligament of the urinary bladder by excision of the persisting ligament and rent. This patient had complete resolution of clinical signs at follow-up approximately 16 months later. This report emphasises the importance of identifying the median ligament of the urinary bladder and minimising soft tissue trauma and formation of rents when performing abdominal surgery.

## Data Availability

No data were analysed, and hence, data sharing is not applicable to this article.
